# Interaction of Sesbania Mosaic Virus Movement Protein with VPg and P10: Implication to Specificity of Genome Recognition

**DOI:** 10.1371/journal.pone.0015609

**Published:** 2011-01-05

**Authors:** Soumya Roy Chowdhury, Handanahal S. Savithri

**Affiliations:** Department of Biochemistry, Indian Institute of Science, Bangalore, India; IBMC-CNRS, France

## Abstract

*Sesbania mosaic virus* (SeMV) is a single strand positive-sense RNA plant virus that belongs to the genus *Sobemovirus*. The mechanism of cell-to-cell movement in sobemoviruses has not been well studied. With a view to identify the viral encoded ancillary proteins of SeMV that may assist in cell-to-cell movement of the virus, all the proteins encoded by SeMV genome were cloned into yeast Matchmaker system 3 and interaction studies were performed. Two proteins namely, viral protein genome linked (VPg) and a 10-kDa protein (P10) c v gft encoded by OFR 2a, were identified as possible interacting partners in addition to the viral coat protein (CP). Further characterization of these interactions revealed that the movement protein (MP) recognizes cognate RNA through interaction with VPg, which is covalently linked to the 5′ end of the RNA. Analysis of the deletion mutants delineated the domains of MP involved in the interaction with VPg and P10. This study implicates for the first time that VPg might play an important role in specific recognition of viral genome by MP in SeMV and shed light on the possible role of P10 in the viral movement.

## Introduction

The primary infection of plants caused by the entry of viruses after mechanical damage to the cell wall and plasma membrane is mostly confined to a single cell. Infection spreads to adjacent cells with the help of viral encoded specialised proteins, called movement proteins (MPs) [1]. The cell to cell movement of viruses is a complex and dynamic process which involves functional contribution from many proteins of viral and host origin [2]. A number of RNA viruses have been identified which require an MP and two or more additional proteins for effective cell-to-cell spread of their genetic material. For example, in the case of potyviruses, the coat protein (CP), an RNA helicase (CI), and a helper component-proteinase (HC-pro) are essential for the movement function [3], [4], [5], [6]. In certain other viruses like hordeivirus and potexviruses, three evolutionarily conserved triple gene block (TGB) encoded TGBp1, TGBp2, and TGBp3, proteins are involved in the process [7], [8], [9]. Another important and conserved feature of many MPs is their nucleic acid binding property, required for transport of viral genome from cell to cell [10]. However, the question of specificity of genome recognition for *in vivo* transport with in the host remains mostly. Not much is known about the molecular aspects of cell–to–cell movement in sobemoviruses and the ancillary proteins have not been identified. Earlier studies have shown that the protein encoded by ORF1 of *Rice yellow mottle virus* (RYMV) [11], *Cocksfoot mottle virus* (CfMV) [12] and *Southern cowpea mosaic virus* (SCPMV) [13] is essential for cell to cell movement. Further, ORF1 encoded product of RYMV is implicated as a RNA silencing suppressor [14], [15].

The genome of *Sesbania mosaic virus* (SeMV), a member of *Sobemovirus* genus is 4149 nucleotides in length and encodes four overlapping ORFs. The ORF 1 codes for the MP, ORF 2a for the poly protein 2a, and ORF 2b for the RNA dependent RNA polymerase (RdRp) that is translated by a frame shift mechanism to yield poly protein 2ab. The 3′- proximal ORF codes for the CP which is expressed via a sub genomic RNA [16]. The domain arrangement in poly protein 2a and 2ab was recently shown to be membrane anchor- protease (Pro)- viral protein genome linked (VPg)- 10 KDa protein (P10) – 8 KDa protein (P8) and membrane anchor- Pro- VPg- RdRp respectively [17]. The structure and the assembly of the virus have been described earlier [18], [19], [20], [21]. The Pro, RdRp, VPg and the P8 domains have been functionally characterised and the latter two are shown to be natively unfolded [22], [23], [24], [25]. In an earlier study SeMV MP was shown to interact with SeMV CP via the N terminal domain [26].

In the present study, the viral encoded ancillary proteins that may be essential for the viral movement in addition to ORF 1 encoded MP were identified. For this purpose, gene segments corresponding to all the domains encoded by SeMV were cloned in Matchmaker yeast 2 hybrid (Y2H) system and their interactions with SeMV MP were studied. Two proteins, namely VPg and P10 were shown to interact specifically with SeMV MP, in addition to CP. The domains in MP involved in these interactions were also identified. These observations, for the first time, implicate that the interaction of MP with VPg which is linked to the 5′ end of the viral genome might provide the specificity for the transport of viral RNA from cell to cell and that the interaction of MP with P10, which is an ATPase [23] might fulfil the energy requirement for the movement function.

## Materials and Methods

### Construction of SeMV clones in matchmaker Gal 4 two-hybrid system 3 vectors

The gene sequences, corresponding to the encoded domains of SeMV, namely Pro, VPg, P10, P8, RdRp, CP and MP, were amplified with high fidelity phusion polymerase (New England Biolabs, 240 County Road, Ipswich, MA 01938-2723, USA), using sense and antisense primer corresponding to the 5′ and 3′ terminal region of the genes respectively (Table 1) with full-length SeMV cDNA clone as the template (AY004291). An additional T was introduced at the frame shift site in the sense primer used for the amplification of RdRp to bring the nucleotide sequence in frame. For bait and prey construction, the PCR products were cloned at *EcoR1* sites of pGAD T7 (Pro, P10, P8, RdRp, VPg and CP) vector having the Gal 4 activation domain with a N terminal hemagglutinin (HA) epitope tag and pGBK T7 (MP and MP deletion mutants) vector having Gal 4 DNA binding domain with a N terminal c-Myc epitope tag. All the recombinant clones were confirmed by PCR with T7 sense and gene specific antisense primers (Table 1), followed by DNA sequencing.

**Table 1:** Description of oligonucleotide primers used in study.

NAME	SEQUENCE 5′-3′	DESCRIPTION
MP SenseMP Anti	CCGGCTAGC **GAATTC**ATGATGGTAATGCAAGCTCAGCATACT CCG**GAATTC**GGAGGAGGACATAGCCCT	Primers for amplification of MP gene. *EcoR*I site is denoted in bold and *Nhe*I site is underlined.
E.CP SenseE.CP. Anti	CCGCATATG **GAATTC**ATGATGGCGAAAAGGCTTTCG CCGCATATG **GAATTC**GTTGTTCAGGGCTGAGGC	Primers for amplification of CP gene. *EcoR*I site is denoted in bold and *Nde*I site is underlined
E.MP N35.Sense	CCGCATATG **GAATTC**ATGATGGTATGTGAAGTGGAATTTGAT	Primer for amplification of MP NΔ 35 gene. *EcoR*I site is denoted in bold and *Nde*I site is underlined
EMP-N16.Sense	CCGCATATG **GAATTC**ATGATGGTATTCATTGGTTTTGAGGAC	Primer for amplification of MP NΔ 16 gene. *EcoR*I site is denoted in bold and *Nde*I site is underlined
E.MP-N49.Sense	CCGCATATG **GAATTC**ATGATGGTAGTGAGAGCCCACAACCAA	Primer for amplification of MP NΔ 49 gene. *EcoR*I site is denoted in bold and *Nde*I site is underlined
E.MP.C3. Anti	CCGCATATG **GAATTC**CATAGCCCTTGCAGCTCG	Primer for amplification of MP CΔ 3 gene. *EcoR*I site is denoted in bold and *Nde*I site is underlined
E.MP C-19 Anti	CCG **AAG CTT** GAA TTC CGG ACA CGA ATA GAA GTA TTC	Primer for amplification of MP CΔ19 gene. *HinD*III site is denoted in bold and *EcoR*I site is underlined
VL MP C-38 Anti	CCG **AAG CTT** GAA TTC CGG CCC GTT TTC ACA AGG AGC	Primer for amplification of MP CΔ38 gene. *HinD*III site is denoted in bold and *EcoR*I site is underlined
Y M-VPg SenseY M-VPg Anti	CCG CCG **AAG CTT** GAA TTC ATG ATG GTA ACT CTC CCA CCG GAG CTT CCG CCG **AAG CTT** GAA TTC CGG CTC TTG AGC GTT TTC CCA	Primers for amplification of Vpg domain. *HinD*III site is denoted in bold and *EcoR*I site is underlined
Y M-P10 SenseY M-P10 Anti	CCG CCG AAG CTT GAA TTC ATG ATG GTA ACC GTC GCT GTT GAG AAT CCG CCG **AAG CTT** GAA TTC CGG TTC CTG CTT GTA ATA ACA	Primers for amplification of P10 domain. *HinD*III site is denoted in bold and *EcoR*I site is underlined
2H RdRp Sense2H RdRp Anti	G ACC GTC GCT GTT GAG AAT TTT AAA CTG CCA GC TTA CGA ATC CGC ACC ATA	Primers for amplification of RdRp gene. One extra nucleotide is incorporated at 5′ end to bring the gene in frame when cloned into Matchmaker vectors at *Sma*I site. Extra T underlined is incorporated to express RdRp from ORF 2b
2H P8 Sense2H P8 Anti	G AGT TTA ATC CTT CCA GAG AGA GGT CTT TGT TGG TGG	Primers for amplification of P8 domain. One extra nucleotide is incorporated at 5′ end to bring the gene in frame when cloned into Matchmaker vectors at *Sma*I site.
2H SP sense2H SP anti	G ATG TAT CAT CCG AGC TGC CTC ATT AGA CCT TAA GAG	Primers for amplification of Serine protease domain. One extra nucleotide is incorporated at 5′ end to bring the gene in frame when cloned into Matchmaker vectors at *Sma*I site.

### Y2H interaction assays

The yeast strain AH109, along with the respective clones in bait and pray plasmid pGAD T7 and pGBK T7 from matchmaker Gal 4 two hybrid system (Clontech Laboratories, Inc. 1290 Terra Bella Ave. Mountain View, CA, 94043, U.S.A), providing HIS3, ADE2, MEL 1 and lac Z reporter constructs and allowing high stringency Y2H selection were transformed and colonies were grown according to the *Yeast Protocols Handbook* ((PT3024-1, Clontech, Heidelberg, 2001). The cells were grown in minimal medium (0.67% yeast nitrogen base, 2% glucose) with appropriate amino acid omission (-Leu and –Trp for yeast transformed with both bait and pray plasmid). Replica plating was performed under conditions of increasing stringency according to the manufacturer's suggestions and subsequently analysed for growth on –Leu/-Trp/-His, -Leu/-Trp/-His/-Ade synthetic drop out (SD) media. The replica plates were analysed with 5-Bromo-4-Chloro-3-indolyl α-D-galactopyranose (α-XGal) to monitor MEL1 expression. Images were captured after 4–6 days of growth at 30°C.

To determine the strength of protein-protein interactions, β-galactosidase solution assay was performed using ortho-nitrophenyl-β- galactopyranoside as a substrate (ONPG; Sigma-Aldrich, 3050 Spruce St., St. Louis, MO 63103, USA). The transformed cells grown in 10 ml of -Leu –Trp –His medium or - Leu –Trp galactose medium (for transformants which failed to grow in the former medium) for 3 to 4 hrs at 30°C were lysed and assayed for β-galactosidase activity. The ß-galactosidase (ß-Gal) activity was quantitated using the following formula: 1,000×[OD_420_−(1.75)]/(*T*×*V*×OD_600_), where optical density at 420 nm (OD_420_) is due to the product formed, OD_600_ is the cell density of the culture, *T* is the reaction time in minutes, and *V* is the volume in ml. The assays were performed in triplicate. Proteins were also isolated from the transformed colonies to quantitate the expression of interacting proteins by ELISA. The proteins (Pro, P10, P8, RdRp, VPg and CP) expressed from pGAD T7 vector were quantified using HA polyclonal antibody as primary antibody, where as MP and the deletion mutants of MP expressed from pGBKT7 were quantified using cMyc monoclonal antibodies. For this purpose, the lysate of cells from 5ml liquid culture grown in appropriate SD medium (-Leu/-Trp/-His) was used for coating the wells.

### Construction and expression of GST-MP clones in *E.coli*

SeMV MP gene was amplified from the full length SeMV cDNA clone as described in the previous section. In order to express MP with N terminal- GST-tag, the PCR product was also cloned at *EcoRI* site of pGEX 4T1 vector (GE Healthcare Bio-Sciences AB,SE-751 84 Uppsala, Sweden). The recombinant clones were confirmed by PCR with T 7 sense and MP antisense primers (Table 1), followed by DNA sequencing.

MP deletion mutant genes were amplified with high fidelity phusion polymerase using sense and antisense primers corresponding to the N and C termini of each of the mutant (Table 1) and with pGEX-MP clone as the template. The PCR products were cloned at *EcoRI* site of pGEX 4T1 vector by site directed ligation. All the clones were confirmed by PCR with T7 sense and MP mutant specific antisense primers (Table 1), followed by DNA sequencing.

pGEX 4T1-MP clone was transformed into *E.coli* BL21(DE3) pLysS cells (Novagen, Merck KGaA, Darmstadt, Germany). A single colony was inoculated into 20 ml of Luria-Bertani (LB) medium containing 50 µg/ml ampicillin and allowed to grow overnight at 37°C. The overnight culture was inoculated into 500 ml of Terrific Broth (TB) containing 50µg/ml ampicillin and allowed to grow at 37°C till the optical density (OD) at 600 nm reached 0.6. The expression of MP was induced with 0.3 mM isopropyl-ß-D- thiogalactopyranoside (IPTG) (Sigma) and grown for 10 h at 15°C. The cells were harvested by centrifugation and resuspended in 30 ml of lysis buffer. The resuspended cells were sonicated for 15 min at an amplitude of 30 (Vibra cell, Sonics & Materials, Inc. 53 Church Hill Road,Newtown, CT 06470-1614 USA) and the lysate was centrifuged at 10,000 g (Avanti JE, Beckmen coulter) for 10 min at 4°C. The solubility of the expressed protein was checked using sodium dodecyl sulphate- polyacrylamide gel electrophoresis (SDS-PAGE) [27]. The protein bands were visualized by staining with coomassie blue (Sigma).

GST-MP was purified from the soluble fraction obtained after sonication of *E.coli* BL21(pLysS) cells expressing GST-MP from the pGEX-MP clone by affinity chromatography using GST binding resin as described in the manufacturers protocol. The purified protein was extensively dialyzed against storage buffer [50 mM Tris HCl, 100 mM NaCl, 10 mM β-marcaptoethanol, (pH 8) and 10% glycerol] to remove the reduced glutathione and stored at −20°C. The same procedure was used for purification of GST-MP deletion mutants. The VPg over expressed in *E. coli* was purified as described earlier [22].

### Electrophoretic Mobility Shift Assay (EMSA)

SeMV was purified from infected *Sesbania grandiflora* leaves 21 days after inoculation and genomic RNA was isolated as described earlier [16].

Fixed concentration of nucleic acid (1 µg) (genomic RNA from SeMV, genomic RNA from *Physalis mottle virus* ( PhMV)) was purified as described earlier [28] , single stranded DNA from SV40 (Bangalore Genei, India), Double stranded DNA (PCR product of SeMV CP gene) were incubated with increasing concentrations of GST-MP or its mutants in incubation buffer [200 mM 3-(N-morpholino)propanesulfonic acid (MOPS), 20 mM sodium acetate pH 7] at 4°C for 30 min. After incubation, the samples were loaded on to 0.5% agarose gel in MOPS electrophoresis buffer [200 mM MOPS, 20 mM sodium acetate pH 7] with 1 µl RNA loading dye (0.25% bromophenol blue, 0.25% xylenecyanol, 30% glycerol in DEPC treated water) and 1 µl ethidium bromide (1 mg/ml) and run for 4 hours at 5 V/cm. After electrophoresis, image of the gel was captured under UV with a gel doc (Gel Doc 1000, Bio-Rad Laboratories, 1000, Alfred Nobel Drive, Hercules, CA 94547).

### ELISA for monitoring *in vitro* protein- protein interaction

The interaction between GST-MP and VPg or P10 *in vitro* was tested by an enzyme-linked immunosorbent based assay (ELISA), as described previously with minor modifications [29], [30], [31], [32]. The ELISA plate (F8 Nunc Maxisorp loose,Nunc, Kamstrupvej 90, Postbox 280, DK-4000, Roskilde, Denmark) wells were coated with 5.0 µg of purified VPg or P10 (100 µl /well) for 1h at 37°C. The protein was diluted in 1× Phosphate-buffered saline (PBS) (pH 7.4). The unadsorbed protein fraction was removed and wells were blocked with 10% skimmed milk in 1× PBS for 1 h at 37°C. The plates were then incubated with GST-MP for 2 h at 37°C. BSA was used as negative control. The wells were washed thrice; 3 min each with 1× PBS containing 0.05% Triton X 100 (PBST) and then thrice with 1× PBS. Polyclonal antibodies specific to the GST-MP (1∶5000) was added and incubated for 1 h. at room temperature. Washes were repeated as before and the wells were further incubated with goat anti rabbit secondary antibody conjugated to HRP (1∶10,000) in 5% skimmed milk in 1× PBS (100 µl /well) for 45 min followed by washing and addition of 1× substrate TMB/H_2_O_2_ (diluted from 20× stock solution in distilled water) and monitored for the blue colour development. The reaction was stopped by the addition of 2 N H_2_SO_4_ (50 µl /well). Interactions were quantified by reading the absorbance at 450 nm using a SpectraMax 340PC384 absorbance microplate reader (Molecular Devices, Inc., 1311. Orleans Drive, Sunnyvale, CA 94089-11361, USA,). All the experiments were done in triplicate and standard deviation was calculated. GST was used instead of GST-MP to rule out possibility of interaction between VPg and GST.

### Thin Layer Chromatography (TLC) and ATPase Assay

To test for ATPase activity, GST MP was incubated with 0.1 µCi [γ^32^P]-ATP at 25°C in the reaction buffer (50 mM MOPS buffer, pH 7.0, 2 mM MgCl_2_, 2 mM dithiothreitol and 0.5 mg/ml of bovine serum albumin). Following incubation for 30 min, the reaction was stopped by adding EDTA (pH 8.0) to a final concentration of 10 mM. Buffer alone and calf intestine alkaline phosphatase (CIAP) were used as negative and positive controls, respectively. The Pi released was detected by TLC on polyethyleneimine-cellulose F plates (obtained from Merck, Germany). The solvent used was 1 M formic acid, 1mM EDTA (pH 8.0) and 0.5 M lithium chloride. The intensity of the spot was measured using phosphor imaging with image gauge software.

## Results and Discussion

### Y2H Study for identification of ancillary proteins that interact with SeMV-MP

**Figure 1:**
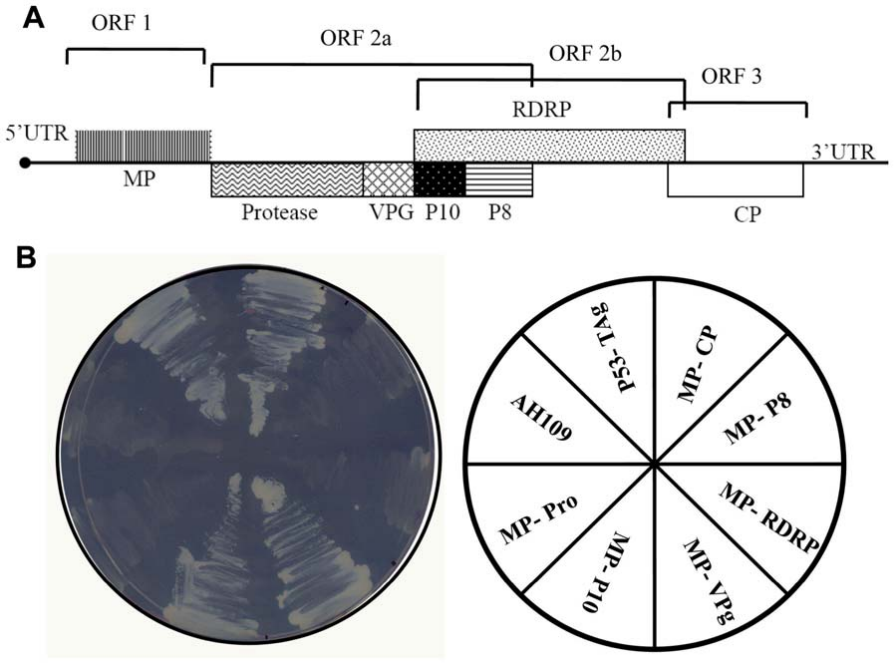
Genomic organization of SeMV and Y2H assay for interacting partner of MP. (A) A schematic representation of the genomic organization of SeMV, representing all the proteins encoded. ORF 1 codes for MP, ORF 2a for poly-protein 2a, ORF 2b for RdRp which is translated by a frame shift mechanism. ORF 3 codes for CP (B) Y2H interaction between MP and all the proteins encoded by SeMV. –Leu –Trp –His plate streaked with AH109 cells transformed with bait and pray plasmids (as shown in the pie diagram). Growth on the plate represents activation of the reporter gene by interaction between the proteins.

The association of ancillary proteins with MPs presumably affect local and systemic spread of the virus within the host. In order to identify viral encoded ancillary proteins that could interact with SeMV MP and may assist in cell to cell movement, all the genes encoded by SeMV genome (Figure 1A) were cloned in matchmaker Gal 4 two hybrid system 3. The Pro, VPg, P10, P8, CP and RdRp PCR products were cloned into pGADT7 and wild type and deletion mutants of MP were cloned into pGBKT7 as described in the methods section. The recombinant pGADT7 and pGBKT7 clones were co-transformed into *Saccharomyces cerevisiae* AH109 strain in pairs as shown in figure 1B. pGBKT7-P53 (Murine p53 fused to GAL4 DNA-BD) and pGADT7-T Antigen (SV40 large T-antigen fused to GAL 4 DNA-AD) were used as positive control in these experiments, as p53 and T Antigen have been shown to interact in a yeast 2 hybrid assay earlier [33]. The growth was monitored on SD medium (SD) not containing Leu and Trp (–Leu –Trp) plates to ensure both the plasmids were co transformed into the yeast cells. The transformed colonies were replica plated on –Leu –Trp -His (medium stringency) plate to determine the interaction, if there is any, between the proteins. As shown in figure 1B, growth of AH109 cells was observed when p53-T Ag, CP-MP, P10-MP, VPg-MP were expressed together. On the other hand, the transformed yeast cells did not grow when P8-MP, RdRp-MP and Pro- MP were co expressed. The results confirmed earlier observations that MP interacts with CP and showed that MP might also interact with VPg and P10 but not with P8, RdRp or Pro domains.

**Figure 2:**
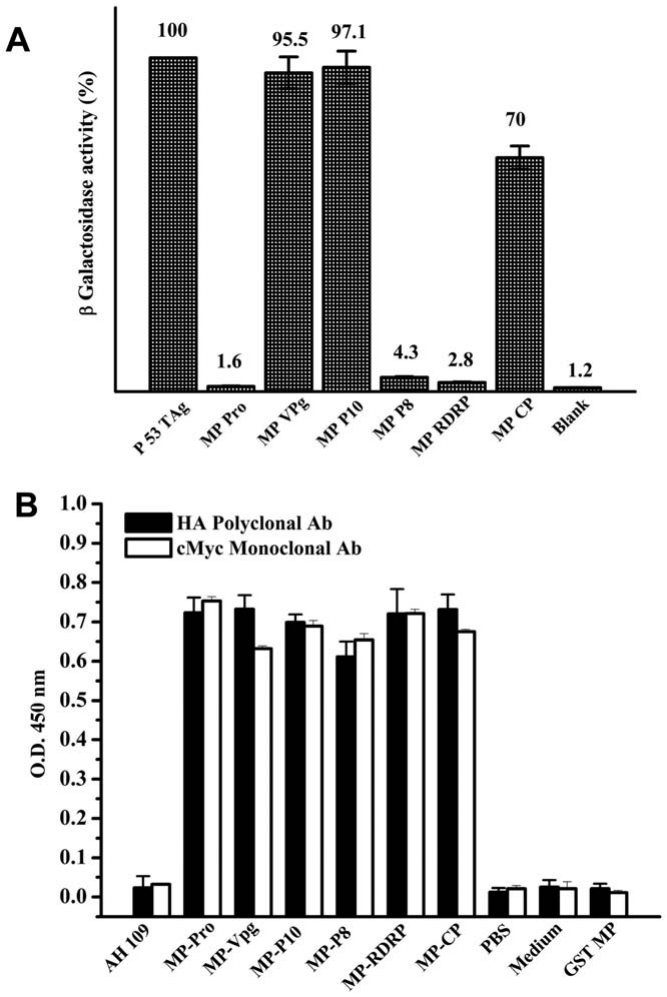
Quantitation of Y2H interaction by ß-galactosidase assay and estimation of level of protein expression. (A) The transformed colonies which showed positive Y2H interaction in the –Leu- Trp- His SD plates were grown in liquid cultures (-Leu –Trp –His medium) and were assayed for β-galactosidase activity to validate and quantify the two-hybrid interactions. For testing the interactions in the pairs that did not grow in -Leu –Trp –His SD plate, the cells grown on -Leu –Trp medium were used. The assay was performed as described in the methods section. The results are presented as the percentage in arbitrary units of ß-galactosidase activity (the values are indicated on the *top* of each *bar*) obtained for the interaction between p53 and TAg (100%). The values represent the mean of at least three separate experiments. (B) ELISA to check the expression of the proteins was performed by coating the total protein isolated from AH109 cells transformed with bait and pray plasmid and using c-Myc monoclonal or HA polyclonal antibody as the primary antibody. The bar represents the absorbance measured at 450 nm for each of the protein pairs. The level of expression of all the HA tagged SeMV proteins was comparable to the expression of cMyc tagged MP.

The transformed colonies which showed positive Y2H interaction were grown in liquid cultures (-Leu –Trp –His medium) and were assayed for β-galactosidase activity to validate and quantify the two-hybrid interactions. For testing the interactions in the pairs that did not grow in -Leu –Trp –His medium, the cells grown on -Leu –Trp medium were used. A single colony was picked from each of the selection medium plate and β Galactosidase assay was performed as described in the methods section. The results obtained are presented as percentage of arbitrary units of ß-galactosidase activity (the values are indicated on the top of each bar) obtained for the interaction between p53 and TAg (100%). The values represent the mean of at least three separate experiments (Figure 2A). The measurement of β-galactosidase activity in these transformants confirmed that MP interacts with CP, VPg and P10 but not with P8, RdRp or Pro. Activity measurements showed that the interaction between MP and VPg or P10 was nearly as strong as that of p53 and TAg (95.5 and 97.1% respectively); where as interaction of MP with CP was weaker with only 70% of the positive control. To ascertain whether the lack of interaction between MP and P8, RdRp or Pro domains was not due to the lack of expression of the proteins in the yeast cells, the expression of all the proteins, both from pGAD T7 and pGBK T7 clones was monitored using HA polyclonal and cMyc monoclonal antibodies respectively using the lysed transformed AH109 cells grown in -Leu -Trp -His medium. As can be seen from figure 2B, the expression level of all the HA epitope tagged SeMV proteins was comparable to the expression of cMyc tagged MP.

### MP-VPg Interaction

VPg is covalently linked to the 5′ end of some viral genomes. VPg acts as a primer for replication by interacting with viral RdRp and also regulates translation by interacting with initiation factors [34], [35]. However, the direct role of VPg in viral movement has not been clearly demonstrated thus far. Recently, SeMV, *Potato virus Y* (PVY, genus *Potyvirus*) and *Potato virus A* (PVA, genus *Potyvirus*) VPgs were reported to be “natively unfolded proteins” [22], [36], [37]. Such proteins possess a number of crucial biological functions including molecular recognition and regulation [38], [39], [40]. The functional diversity provided by disordered regions is believed to complement functions of ordered protein regions by protein-protein interactions [41]. Therefore we wanted to probe this unique MP-VPg interaction further and determine its possible functional significance.

**Figure 3:**
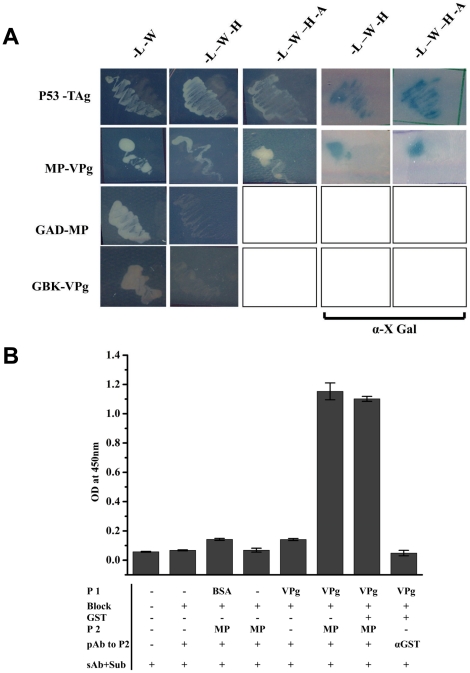
Y2H and ELISA based assays for interaction between MP and VPg. (A) Y2H interaction between MP and VPg. pGBK T7 (MP and p53 ) and pGAD T7 (VPg and T Ag) clones were transformed in pairs into AH109 strain and plated on to –Leu-Trp SD transformation selection plates and incubated for 96 hrs. Colonies which grew were marked and again replica plated onto various nutritional marker SD plates having different stringency of reporter gene expression as shown in the figure. To determine α- galactosidase activity, colonies were plated onto SD plates having α-X Gal (last two columns). AH109 cells transformed with pairs of pGBK T7-MP and pGAD T7 or pGAD T7-VPg and pGBK T7 clones were also plated on to selection plates to rule out the possibility of auto activation of reporter genes (last two rows). (B) ELISA based interaction study between GST-MP and VPg. VPg (5 µg) (P1) coated ELISA plates were blocked with 10% skimmed milk in 1× PBS (Block) followed by the addition of 5 µg of GST-MP (P2), ELISA was performed with anti MP polyclonal antibody (pAb to P2) and developed using anti rabbit IGg and DMB H_2_O_2_ (sAb+Sub). Details of the steps and controls are marked in the figure; BSA and GST were used as the negative controls. The bar represents the absorbance of the samples measured at 450 nm.

pGBK T7 MP and pGAD T7 VPg were retransformed into AH109 strain, and plated onto SD –Leu –Trp plates and incubated for 96 hrs. The transformants were marked and transferred to higher stringency plates as described earlier. As shown in Figure 3A, the transformants could grow at highest stringency level (–Leu –Trp –His –Ade +α X Gal plates) suggesting the activation of all the reporter genes to the same extent as was observed with p53 and TAg interaction. It was also ascertained that there was no auto activation of the reporter genes by the presence of proteins alone, as VPg or MP expressed independently along with the pGBK T7 or pGAD T7 vector alone respectively in AH109 cells showed no growth on the selection medium plates. These results clearly establish that MP interacts with VPg.

Next, purified GST-MP and VPg were used for checking their interaction *in vitro* by ELISA, where, VPg (5µg) was immobilised on to the ELISA plates, and blocked with 10% skimmed milk in PBS followed by addition of GST-MP (5µg), which was used as the probe protein. The interaction between the two proteins was monitored by anti MP polyclonal antibodies. As shown in Figure 3B, a significant absorbance at 450 nm due to the interaction of MP with VPg was observed. In the experiment BSA and GST were used as negative controls. Further, cross reaction of the primary antibody to the immobilized protein, i.e. MP polyclonal antibody to VPg or the milk proteins used for blocking were also tested by ELISA in the absence of MP. From the results it can be concluded that MP interacts with VPg *in vitro* also.

### Nucleic acid binding studies of SeMV MP

**Figure 4:**
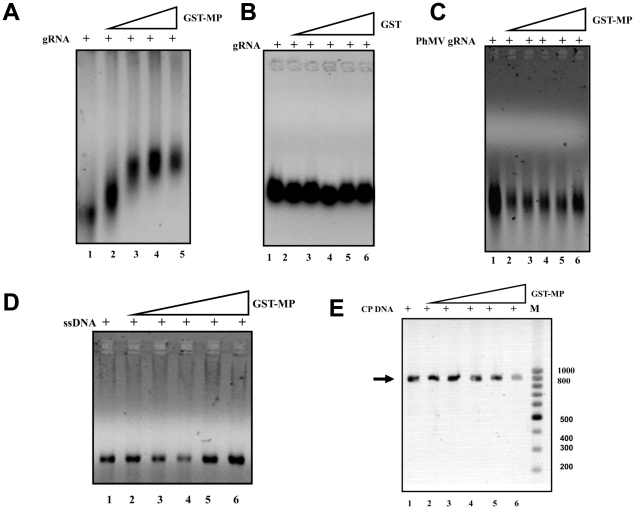
Electrophoretic mobility shift assay for binding of nucleic acid substrates to GST-MP. (A) GST-MP (1, 2, 3 and 4 µg lanes 2–5) was incubated with SeMV genomic RNA (1 µg) in the incubation buffer [200 mM MOPS, 20 mM sodium acetate pH 7] at 4°C for 30 min. The nucleoprotein complex was visualized after electrophoresis on a 0.5% agarose gel, followed by staining with ethidium bromide. Lane 1-RNA alone. (B) Purified GST (1, 2, 3, 4 and 5 µg lanes 2–6) was incubated with SeMV genomic RNA (1 µg) in incubation buffer (as above) at 4°C for 30 min and EMSA was performed as above. Lane 1-RNA alone. (C) Purified GST-MP (1, 2, 3, 4 and 5 µg lanes 2–6) was incubated with PhMV genomic RNA (1 µg) in incubation buffer at 4°C for 30 min and analyzed. Lane 1-RNA alone. (D) GST-MP (1, 2, 3, 4and 5 µg lanes 2–6) was incubated with M13 ssDNA (1 µg) in incubation buffer (as above) at 4°C for 30 min and analysed as before. Lane 1-ssDNA alone. (E) GST-MP (1, 2, 3, 4 and 5 µg lanes 2–6) was incubated with SeMV CP gene PCR product (1 µg) in incubation buffer (as above) at 4°C for 30 min. The nucleoprotein complex was visualized after electrophoresis on a 0.5% agarose gel, followed by staining with ethidium bromide. Lane 1-dsDNA alone.

Many of the well characterised MPs bind to nucleic acids. Therefore, the nucleic acid binding property of purified GST-MP was investigated. Increasing concentration GST-MP was incubated with a fixed concentration of viral RNA purified from SeMV and the complex was analysed on 0.5% agarose gel and photographed under UV (Figure 4A). The mobility shift observed with increasing concentration of GST-MP confirms the ability of the protein to bind to genomic RNA. To rule out the possibility of GST- RNA interaction, EMSA was performed with genomic RNA and similarly expressed and purified GST (Figure 4B). No interaction was observed confirming that the mobility shift observed in the gels (Figure 4A) was a result of MP-RNA interaction.

Interestingly, no interaction was observed when GST-MP was incubated with other nucleic acids such as PhMV genomic RNA (Figure 4C), M13 ssDNA (Figure 4D), and dsDNA in the form of SeMV CP gene PCR product (Figure 4E). These results demonstrate that SeMV MP can only recognize its own genomic RNA. It may be noted that only SeMV genomic RNA has covalently linked VPg. On the other hand, studies with MPs from different genera have shown that, they have diverse nucleic acid binding properties. For example, *Red clover necrotic mosaic virus* MP binds to both ssRNA and ssDNA, [42], *Cocksfoot mottle virus* P1 protein can interact with ssRNA transcripts in a sequence-non-specific manner [43], while *Bean dwarf mosaic virus* transports ssDNA and not ssRNA or dsDNA [44]. This unique feature of SeMV-MP with specificity towards its own genome was investigated further.

### Does MP recognize genomic RNA through interaction with VPg?

The results presented thus far clearly demonstrated that SeMV MP interacts with VPg and it can also bind to genomic RNA specifically. Two possible explanations for the specific binding of SeMV MP to the genomic RNA are- i) sequence specific interaction of MP with the genome or, ii) direct interaction of MP with the VPg covalently linked to the 5′end of the RNA.

**Figure 5:**
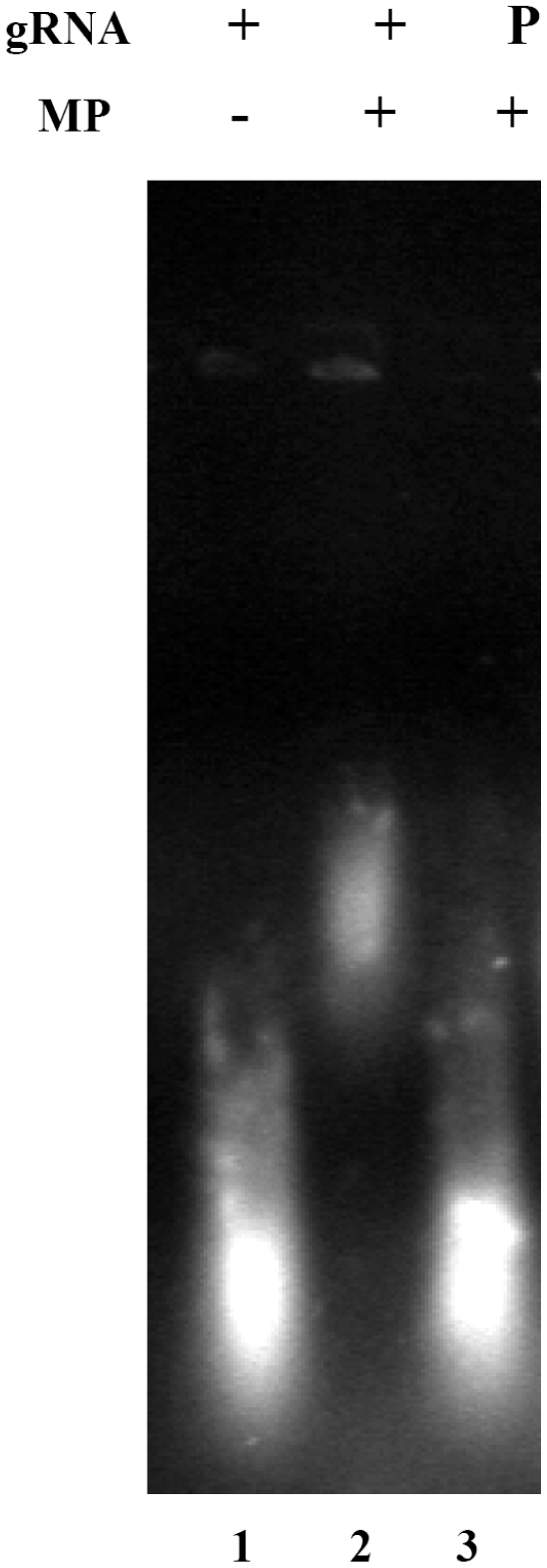
Role of VPg in the recognition of genomic RNA by MP. Purified GST-MP (5 μg) was incubated with genomic RNA (1 μg) or pronase treated and trizol purified genomic RNA (1 μg) in incubation buffer [200 mM MOPS, 20 mM sodium acetate pH 7] at 4°C for 30 min. The nucleoprotein complex was visualized after electrophoresis on a 0.5% agarose gel, followed by staining with ethidium bromide. Lane 1- genomic RNA alone, Lane 2- genomic RNA with GST-MP and Lane 3- pronase treated genomic RNA with GST-MP.

To test these possibilities, *in vitro* transcripts of the CP gene, which lacked the covalently linked VPg at the 5′ end, were made and EMSA was performed with GST-MP. It was observed that MP was unable to bind to the *in vitro* transcribed RNA (data not shown). Next, the genomic RNA of SeMV was treated with Pronase {Protease from *Streptomyces griseus*, Sigma Aldrich, [45]} to remove the VPg and the RNA was purified by trizol method as described earlier [28]. When EMSA of the Pronase treated RNA was performed with GST-MP as described previously, it was observed that GST MP was unable to recognize genomic RNA without VPg (Figure 5). To ascertain the presence of VPg covalently linked to the genomic RNA, ELISA was performed with 0.5 µg of genomic RNA samples with polyclonal antibodies to VPg or CP as primary antibody (data not shown). The well in which genomic RNA was coated gave signal at 450 nm when VPg antibodies were used and not when CP antibodies were used, thus ruling out the possibility of MP-RNA interaction via CP that might have co-purified with the genomic RNA. These results, demonstrate that SeMV MP interacts with genomic RNA via the covalently linked VPg. Such an interaction could facilitate specific viral RNA movement from one cell to another. In the case of potyviruses, which also have covalently linked VPg at the 5′ end of their genome, it has been demonstrated that VPg interacts with host encoded VPg interacting proteins (PVIP) and assists in cell to cell movement [46].

### Generation of SeMV MP deletion mutants for the identification of domains necessary for interaction with the ancillary proteins

Further experiments were carried out to delineate the regions in SeMV MP which may be involved in the interaction with the ancillary proteins. MP is primarily a α helical protein as predicted from the amino acid sequence. The predicted N-terminal three helices were deleted one at a time to obtain NΔ16, NΔ35 and NΔ49 mutant MPs. From the C terminus three serine residues were deleted to generate CΔ3 mutant. The stretch of three serine residues was predicted to have a high propensity for phosphorylation. Previously it was reported in literatures that some of the MPs are regulated by phosphorylation / dephosphorylation as has been shown for *Tobacco mosaic virus* (TMV), *Tomato mosaic virus* (ToMV) and *Potato leafroll virus* (PLRV), [47]. CΔ19 deletion mutant was created to remove a predicted nucleic acid binding domain. Deletion of 38 amino acids from the C-terminus resulted in the deletion of three of the cysteine residues and the nucleic acid binding domain. The deletion mutants were constructed by PCR amplification with appropriate sense and anti sense primers (table 1). The PCR products were cloned into pGBK T7 vector at the *EcoRI* site to obtain bait constructs with Gal 4 DNA binding domain as described earlier for full length MP. The same mutants were also cloned into pGEX 4T1 vector at the *EcoRI* site as described earlier. These mutants were over expressed in *E.coli* BL21 pLys S cells. All the GST-MP deletion mutants were soluble and were of expected sizes. The proteins were purified using GSH affinity chromatography and used for *in vitro* experiments.

### Identification of domains of MP necessary for interaction with VPg

MP mutants in pGBK T7 were transformed with pGAD T7 VPg into AH109 cells to map the domains in MP which could be important for interaction with VPg. Transformation was carried out as described earlier.

**Figure 6:**
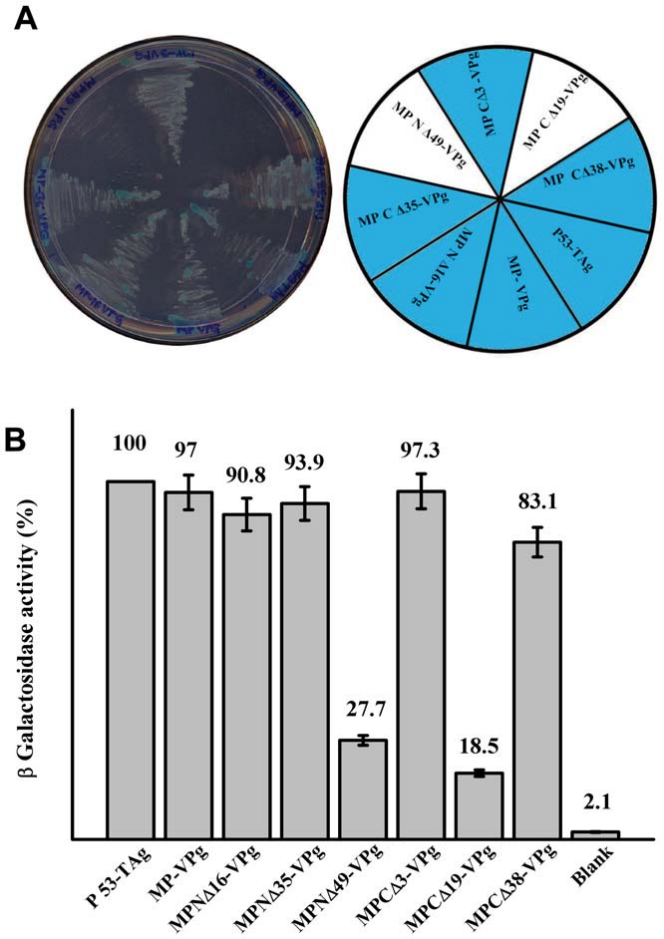
Determination of the domains in MP necessary for interaction with VPg by Y2H interaction. (A) pGBK T7 (MP, MP deletion mutants and p53 ) and pGAD T7 (VPg and T Ag) clones were transformed in pairs into AH109 strain and plated on to –Leu-Trp –His –Ade+α XGal SD high stringency reporter selection plates and incubated for 96 hrs in the dark. Transformants are marked in the pie diagram. (B) Quantification of interaction of MP and MP deletion mutants with VPg by ß-Galactosidase assay. ß-Galactosidase assay of transformed colonies which showed positive Y2H interaction on –Leu –Trp –His –Ade plates was preformed as described in the materials and method section. Data are presented as the percentage of arbitrary units of ß-galactosidase activity (the values are indicated on the *top* of each *bar*) with respect to the interaction between p53 and TAg (100%). The values represent the mean of at least three separate experiments.

As is evident from figure 6A, the interaction of MP with VPg was abolished at the highest stringency upon deletion of the N terminal 49 and C terminal 19 amino acids of MP. No colonies appeared in –Leu –Trp –His -Ade+X-Gal plates when these two pGBKT7 NΔ49 and CΔ 19 MP mutants were transformed with pGAD T7 VPg separately. Interaction between all the other mutants of MP with VPg produced blue colonies. Interestingly, even CΔ38 produced blue colonies. β–galactosidase activity of these transformants also corroborated with these results (Figure 6B). As evident, VPg- MP N Δ49 and VPg MP CΔ19 showed 27.7% and 18.5% activity when compared with that obtained with p53-TAg interaction. In conformity with the results presented in figure 6A, VPg- MP CΔ38 interaction resulted in 83.1% ß- galactosidase activity. It is possible that the deletion of C terminal 19 amino acids affects proper folding of MP, thereby preventing interaction with VPg. However deletion of C terminal 38 amino acids may restore the core structure of MP which might favour interaction with VPg. ELISA of the transformed AH109 cells showed that the level of proteins expressed from both the vectors remained nearly the same, confirming that the reduced level of activity was due to loss of interaction between MP NΔ49, MP CΔ19 with VPg. As explained earlier, the loss of interaction with MP CΔ19 could be due to improper folding of this mutant protein. These results demonstrate that the interaction of SeMV-MP with VPg is primarily through its N terminal 49 amino acids residues.

### Interaction of SeMV MP deletion mutants with genomic RNA

**Figure 7:**
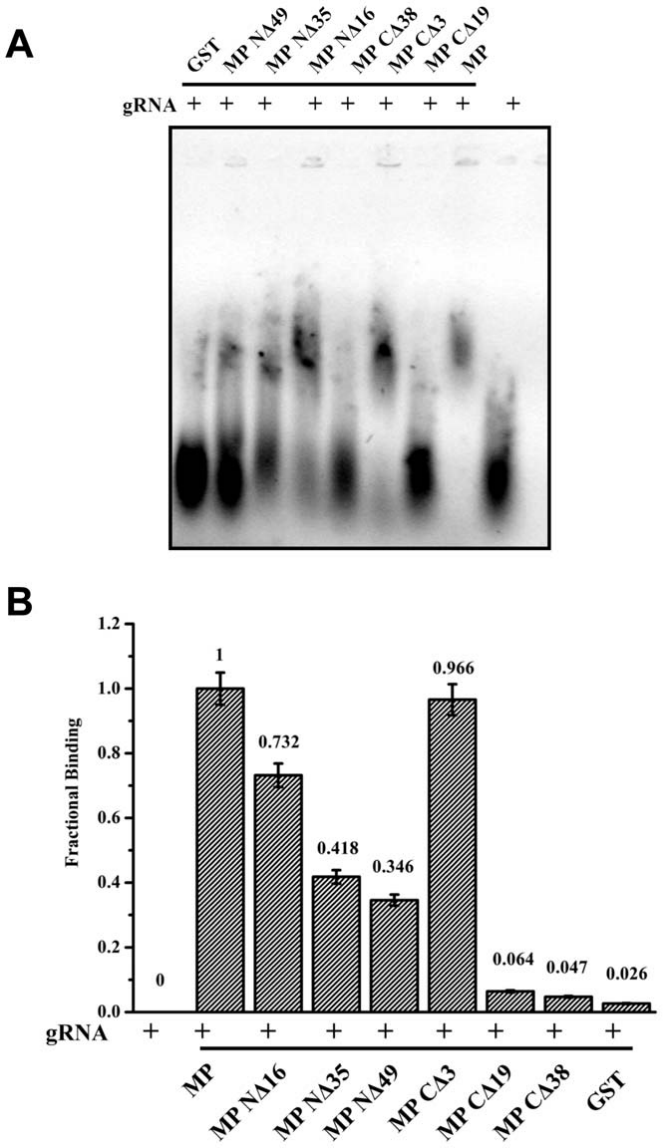
EMSA of MP and its deletion mutants with viral RNA. **(A)** Purified GST, GST MP NΔ49, GST MP NΔ35, GST MP NΔ16, GST MP CΔ38, GST MP CΔ19 and GST MP (5 µg each) were incubated with genomic RNA (1 µg) in incubation buffer [200 mM MOPS, 20 mM sodium acetate pH 7] at 4°C for 30 min. The nucleoprotein complex formed was analysed along with genomic RNA not incubated with MP or its mutants by electrophoresis on a 0.5% agarose gel, followed by staining with ethidium bromide. (B) Fractional binding of RNA was calculated and plotted as a bar diagram for all the proteins using the formula, Fractional binding = Protein bound RNA/ Total RNA.

Since the MP recognizes genomic RNA via interaction with VPg, the mutants which are unable to bind or recognize VPg may not be able to form RNP complex with the genomic RNA. To test this hypothesis, EMSA was performed with GST-MP and its deletion mutants with genomic RNA (Figure 7A) and the fractional binding was calculated by measuring the intensity of the band corresponding to genomic RNA and RNA protein complex and using the formula, Fractional binding = Protein bound RNA/ Total RNA (Bound+Free) as shown in Figure 7B.

As can be observed MP NΔ49, MP CΔ 19 and MP CΔ 38 were unable to form complex with the genomic RNA. The lack of binding of viral RNA could be easily explained for the former two mutants as these mutants failed to interact with VPg in the Y2H study (Figure 6). However, although MP CΔ 38 interacted with VPg (Figure 6) it did not show the mobility shift with genomic RNA. It may be noted that deletion of C terminal 19 and 38 amino acids of MP results in the loss of predicted RNA binding domain. These results suggest that the initial interaction of MP with genomic RNA is mediated by interaction with VPg. This interaction might trigger conformational changes in MP that allows the RNA binding domain of MP to interact with RNA.

### MP- P10 interaction

**Figure 8:**
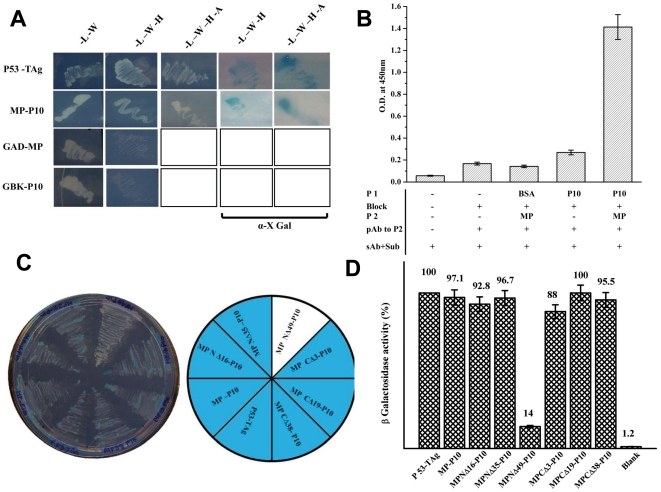
MP-P10 interaction by Y2H assay. (A) pGBK T7 (MP and p53 ) and pGAD T7 (P10 and T Ag) clones transformed in pairs into AH109 strain and plated on to –Leu-Trp SD transformation selection plates and incubated for 96 hrs. Colonies which grew were marked and again replica plated on to various nutritional marker SD plates as shown in the figure. To ascertain α- galactosidase activity, colonies were plated onto SD plates having α-X Gal. AH109 cells transformed with pairs of pGBK T7-MP and pGAD T7 or pGAD T7-P10 and pGBK T7 clones were also plated on to selection plates to rule out the possibility of auto activation of reporter genes (last two rows). (B) ELISA based interaction study between GST-MP and P10. P10 (5 µg) (P1) coated ELISA plates were blocked with 10% skimmed milk in 1× PBS (Block) followed by the addition of 5 µg of GST-MP (P2), ELISA was performed with anti MP polyclonal antibody (pAb to P2) and developed using anti rabbit IGg and DMB H_2_O_2_ (sAb+Sub). Details of the steps and controls are marked in the figure; BSA was used as the negative control. The bar represents the absorbance of the samples measured at 450nm (C) Determination of the domains in MP necessary for interaction with P10 by Y2H interaction. pGBK T7 (MP, MP deletion mutants and p53 ) and pGAD T7 (P10 and T Ag) clones were transformed in pairs into AH109 strain and plated on to –Leu-Trp –His –Ade +α XGal SD high stringency reporter selection plates and incubated for 96 h in the dark. Transformants are marked in the pie diagram. (D) Quantification of MP and MP deletion mutants – P10 Y2H interaction by ß-galactosidase assay. ß-galactosidase assay of transformed colonies which showed positive Y2H interaction on SD reporter selection plates was performed as described in the methods section. Data are presented as the percentage of arbitrary units of ß-galactosidase activity (the values are indicated on the *top* of each *bar*) obtained for the interaction between p53 and TAg (100%). The values represent the mean of at least three separate experiments.

As shown earlier, P10 was another protein that interacted with SeMV MP in Y2H experiments. To further characterise the interaction in detail, pGBK T7 MP and pGAD T7 P10 were retransformed in AH109 strain and plated onto SD –Leu –Trp plates and incubated for 96 hrs. The transformed colonies were replica plated again on to SD –Leu-Trp plates to confirm the transformation. The colonies were marked and selected on higher stringency plates as described earlier and are shown in Figure 8A.

MP and P10 were found to interact upto the highest level of stringency i.e., –Leu –Trp –His –Ade +X Gal as evident from the appearance of blue colonies on the plate similar to MP –VPg interaction, signifying a strong interaction between the two proteins. The interaction was specific, as pGBKT7 MP or pGADT7 P10 transformed individually along with pGADT7 or pGBKT7 respectively did not result in the appearance of colonies under any nutritional selection (Figure 8A last two rows).

Interaction of MP with P10 *in vitro* was also tested by ELISA. For this purpose, GST-MP and P10 over expressed in *E.coli* and purified were used in this experiment as described in the methods section. As apparent from figure 8B, plate bound P10 interacted with GST- MP specifically.

### Identification of domains in MP necessary for interaction with P10

MP mutants in pGBK T7 and pGAD T7 P10 were transformed into AH109 strain to map the regions of interaction between MP and P10. As evident from the figure 8C, the interaction between P10 and MP was reduced drastically by the deletion of first 49 amino acids from the N terminus of MP as only white colonies were produced in –Leu –Trp –His -Ade +X Gal plates, while interaction between all the other mutants of MP with P10 resulted in blue colonies. β –galactosidase assay also confirmed the same result (Figure 8D). As evident MP NΔ49- P10 Y2H interaction resulted in only 14% of the ß- galactosidase activity as compared to that observed with p53- T Ag interaction or the other MP deletion mutants with P10. This drastic reduction in the activity in MPNΔ49- P10 expressing cells was not due to the lack of expression of the proteins, since, the level of expression of the two interacting proteins in all the sets remained nearly the same (data not shown). These results show that MP and P10 interact with each other predominantly via the N terminal domain of MP. However, as shown in figure 8C all the transformants could grow on –Lue -Trp-His -Ade it is possible that P10 and MP might have other sites of interaction within the region N 49 to CΔ 38 of MP. It may be noted that all the three ancillary proteins CP (data not shown), VPg and P10 interact with MP via the N terminal domain primarily. It would be interesting to investigate whether or not these interactions are mutually exclusive. The specific interaction of MP with P10 could be of physiological relevance. The P10 domain of SeMV poly protein 2a was recently shown to exhibit ATPase function [23]. The cell-to-cell movement of viruses is an active process and some MPs or their ancillary proteins possess ATPase function. For example, HSP70h from Closterovirus which is involved in virus translocation from cell to cell, has a well characterized ATPase function [48]. Similarly, the TGBpl proteins encoded by distantly related viruses contain conserved sequence motifs of some ATPases and helicases [49], [50], [51]. The PVX 25K MP and its counterparts in *Figwort mosaic virus* and *Barley stripe mosaic virus* have been shown to exhibit ATPase activity [52].

To examine if SeMV MP also exhibited ATPase activity, ATPase assay was carried out at 25°C for 30 min in standard reaction mixtures as described in the methods section. GST- MP was unable to hydrolyse ATP where as calf intestine alkaline phosphatase (CIAP) used as positive control could hydrolyse ATP (data not shown). It is possible that P10 having the ATPase function assists the active movement process of M complex by interaction with MP.

The results presented in this communication clearly demonstrate that SeMV MP interacts with two viral encoded proteins P10 and VPg in addition to CP. The recognition of cognate RNA by MP probably occurs via protein-protein interaction between MP and VPg. Interaction of SeMV MP with P10 might be crucial for the active transport of viral RNA complex, the energy for which could come from the hydrolysis of ATP by P10.
